# Use of a neural network to predict normalized signal strengths from a DNA-sequencing microarray

**DOI:** 10.6026/97320630013313

**Published:** 2017-09-30

**Authors:** Charles Chilaka, Steven Carr, Nabil Shalaby, Wolfgang Banzhaf

**Affiliations:** 1 Program in Scientific Computing; 2 Department of Biology; 3 Department of Computer Science; 4 Department of Mathematics and Statistics Memorial University of Newfoundland; St. John's, Newfoundland, Canada A1C 5S7; 5 Department of Mathematics, FUT, Owerri, Nigeria; 6 Present address: Department of Computer Science and Engineering, Michigan State University, East Lansing MI 48824

**Keywords:** Neural networks, n-grams, Performance, Regression values

## Abstract

A microarray DNA sequencing experiment for a molecule of N bases produces a 4xN data matrix, where for each of the N positions each quartet comprises the signal strength of binding of an experimental DNA to a reference oligonucleotide affixed to the microarray, for the four possible bases (A, C, G, or T). The strongest signal in each quartet should result from a perfect complementary match between experimental and reference DNA sequence, and therefore indicate the correct base call at that position. The linear series of calls should constitute the DNA sequence. Variation in the absolute and relative signal strengths, due to variable base composition and other factors over the N quartets, can interfere with the accuracy and (or) confidence of base calls in ways that are not fully understood. We used a feed-forward back-propagation neural network model to predict normalized signal intensities of a microarray-derived DNA sequence of N = 15,453 bases. The DNA sequence was encoded as n-gram neural input vectors, where n = 1, 2, and their composite. The data were divided into training, validation, and testing sets. Regression values were >99% overall, and improved with increased number of neurons in the hidden layer, and in the composition n-grams. We also noticed a very low mean square error overall which transforms to a high performance value.

## Background

DNA sequences although letters contain a lot of information. They are not numeric in nature but their conversion to numerical values en-ables the application of powerful digital signal processing techniques to them. Some desirable properties of a DNA numerical representation are given in [3]. Some forms of DNA numerical representations include: Z-curves and DNA walks [4], Voss method, quaternion technique and paired nucleotide/atomic number representation [5], paired numeric representation [6], double curve and structural profile method [7] and electron-ion interaction potential [8]. N-gram method used in this paper was first introduced by C.E Shan-non in 1948 [9], and makes use of data in a sliding window fashion and neural network learning methods provide a robust approach to approximating real-valued, discrete-valued and vector-valued target functions [12] like DNA numerical. The study of artificial neural networks has been inspired in part by the observation that biological learning systems are built of very complex webs of interconnected neurons [10, 11, 12], where the neurons communicate through a large set of interconnections with variable strengths (weights) in which the learned information is stored [13]. Each neuron computes a weighted sum of its y input signals. The activation function for neurons is the sigmoid function defined [12] as

δ(y) = 1 / (1+e-y) - (1)

Where y is the weighted sum of the inputs. The output of the sigmoid function ranges from 0 to 1, increasing monotonically with its input and the weights of the interconnections between the different neurons are adjusted during the training process to achieve a desired input/output mapping. The ideas from artificial neural net-works have led to computational analysis of human DNA sequence [14], single base pair discrimination of terminal mismatches [15], biological phenomena through computational intelligence [16], human donor and acceptor sites prediction [17], coding region recognition and gene identification [18], predicting transmembrane domains of proteins [19] and the prediction of nucleotide sequences using genomic signals [20, 21].

In this paper, an Affymetrix [1] experiment output, which has numerical values, is normalized and partitioned into training; testing and validation set using a Matlab [2] neural network with 4 and 16 numbers of nodes in the input layer. The influence of the length of nucleotide in the nucleotide hybridization intensity [22] lets us replace the nucleotide and di-nucleotide sequences with their respective n-gram counts. The n-gram ratios are shown in Table 1 and Table 2.

**Figure 1:**
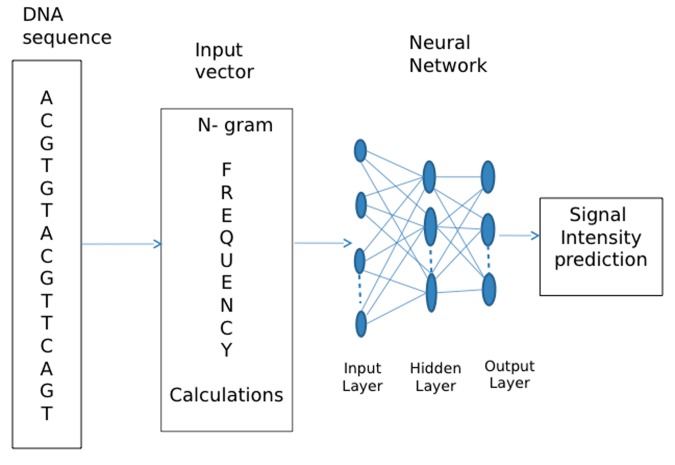
A neural network system for signal intensity prediction. The DNA sequences are first converted into n-gram profiles as input vectors. The neural network then predicts the normalized signal intensities after network training.

Experimentation is done with different number of neurons in the hidden layer that give an optimal prediction performance. The out-put node layer has in our case 4 nodes reflecting our choice of sequence signals to predict. The schematics of DNA neural network architecture are shown in Figure 1. The DNA sequence is first converted by a sequence-encoding schema into neural network input vectors (ratios of n-gram). The neural network then predicts those normalized intensities according to the sequence information embedded in the neural interconnections after network training.

## Methodology

The dataset is adopted from the Cambridge Reference Sequence with ascension number NC--012920 and is made of 15,453 rows and 6 columns where 2 of the columns are the n-grams for n= 1,2 and the other 4 columns represent the normalized intensities for Adenine, Cytosine, Guanine and Thymine. We extract every 26th line of the dataset, which reduces the dataset to 594 rows (lines). We use 1-grams and 2-grams independently to predict the normalized intensities for the four nucleotides ACGT and also use a combination (composition) of the 1-gram and 2-gram to repeat the analysis. The algorithmic steps for our data manipulation are as follows:

1: Compute n-gram profiles of the DNA data set using Python programming language. 2: Calculate the nucleotide and dinucleotide frequencies of these profiles. 3: Do substitution of the nucleotides and dinucleotide strings with their respective frequencies. Do the following on the intensity profiles: 4: Calculate the highest and lowest value along each row. 5: Do normalization along each row using.

N (i) = (yi - min) / (max - min)

**Figure 2:**
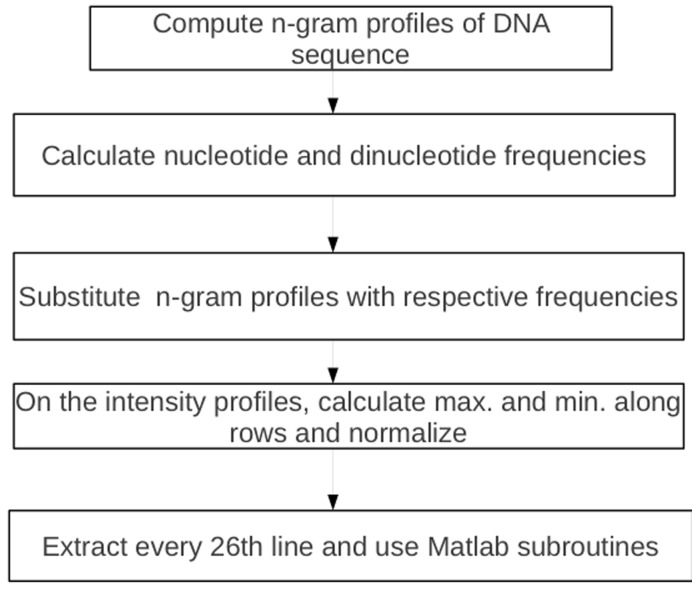
Algorithmic flowchart for computing n-gram profiles and doing normalization on the DNA sequence.

Where yi is the actual value of the attribute max, min and I are the maximum and minimum values along each row. 6: Repeat step 5 for every row of intensity profile. 7: Combine results obtained from step 1 to step 6. 8: Extract every 26th line from the data set after the operations above. 9: Use Matlab subroutines to get performance plots and regression values. The flowchart for the steps is shown in Figure 2.

### Data evaluation functions

In Matlab neural networks, there are functions that help check whether things are consistent. Two of them, which are used in this paper, are to avoid subsequence overlap and possibility of random match

**Figure 3:**
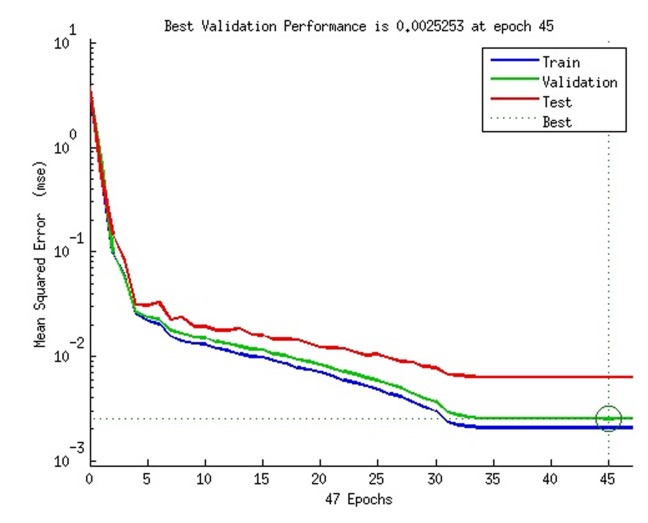
A 1-2 gram composition performance plot with 40 neurons in the hidden layer showing training, validation and testing data set in terms of mean square error.

Performance: This is a plot of the training, validation and test errors. It shows the mean square error MSE dynamics in a logarithmic scale. The training MSE is always decreasing and the least. Validation and test MSE are of more interest and are supposed to be similar for a near perfect training. Training on the data set normally stops when there is a consistent in-crease in the validation error for a given number of iterations. The best performance is taken from the epoch with the lowest validation error. Figure 3 shows a performance plot.

**Figure 4:**
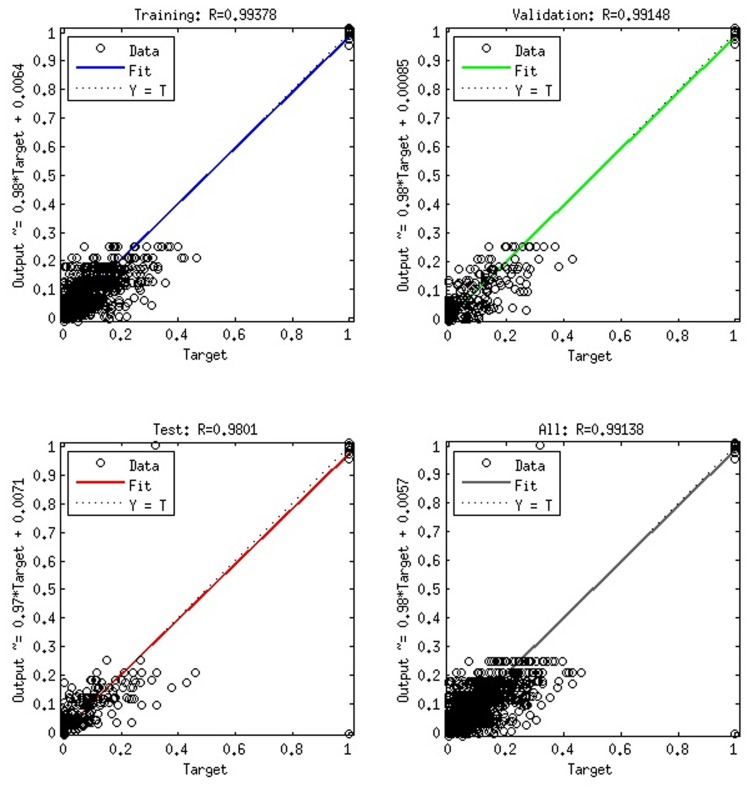
A 1-2-gram composition regression plot with 20 neurons in the hidden layer showing training, validation, testing and overall regression values.

Regression: This performs a linear regression analysis between the network outputs and the corresponding targets. The solid line represents the best-fit linear regression line between outputs and targets. In an ideal situation, i.e. with zero error, the points are placed on the target=output line. High regression values are indication of good results. The scatter plot is helpful in showing that certain data points have poor fits. Figure 4 shows a regression plot.

## Results

The regression value R, so computed by the neural network deter-mines how robust the prediction is. The higher the R values the better and a smaller MSE in terms of performance implies good pre-diction. We compare the performances of the networks with 1-gram and 2-gram with different number of neurons in the hidden layer. The number of neurons in the hidden layer has been varied between 20 and 40 with step size 5 as a matter of choice and hopefully to find the optimal network architecture.

Table 3 gives a summary of the regression values extracted from 1-gram outputs for ACGT and their averages in terms of training, validation and testing using every 26th line (row) with Matlab regression toolkit. The table shows maximum regression values, which corresponded to training set which is consistent with the expected result.

Table 4 gives a summary of the regression values and their averages using 2-gram. The table shows maximum regression values for ACGT again corresponding to the training set.

Table 5 gives a summary of the regression values and their averages using 1-2-gram composition. Again, the table shows the maximum regression values for ACGT corresponding to the training set, which is indicative of a good result.

Table 1 and Table 2 show the percentages (ratios) from Affymetrix [1] dataset of nucleotides and dinucleotides respectively.

## Discussion

The absolute set comprises 4x594 values, where the four values are the absolute signal strengths of the bases [ACGT] on each of 594 lines. Absolute signal strengths are normalized to values between 0.0 -1.0, from which the Neural Network / n-gram process predicts values (≥0.0-1.0). The Prediction set correctly identifies the highest value (1.0) in the normalized set for all 594 lines, which is, of course, the highest value and therefore the correct base call in the absolute set. This is not necessarily a trivial result, as the predictive function must accommodate all targets in the 4 x 594 sets. Using regression toolkit, we observed from Table 3, Table 4 and Table 5 that the best regression values in terms of training, testing and validation were gotten when we used the 1- 2-gram composition with best performance value of 0.002525 with 40 neurons in the hidden layer as shown in Table 5 which translates to 99.9975 % accuracy. This shows a very low mean square error.

## Conclusion

The results of this study show that Artificial Neural Networks based n-gram model for prediction of normalized signal intensities is at least accurate based on high regression numerical values obtained with their attendant low mean square errors which is a measure of performance. Hence, we can use n-gram model to predict the signal intensities via their normalized values from Affymetrix data. The result produced from this research can still be used if one wants to investigate individual nucleotide intensities along a given sequence. We have used mainly 1-gram and 2-gram to carry out analysis. One may improve upon these results if higher n-gram values and their different compositions are considered. An effort could also be made to get optimal number of neurons in the hidden layer that give maximal regression values and lower mean square errors. An increase in regression value to say 0:999 are indicative of a much better prediction. Other forms of normalization like Min-Max, Z-score and normalization by decimal scaling could also be explored to compare results. One can also choose other forms of data evaluation functions in Mat-lab to check if these results are consistent. As a form of confirmation, other forms of numerical representations of DNA sequence mentioned earlier can be used to predict normalized signal intensities recorded by platforms like Affymetrix Genechip and useful comparisons can be made.

## Figures and Tables

**Table 1:** The nucleotide percentages (ratios)

Nucleotides	A	C	G	T
Ratios	0.31	0.31	0.13	0.25

**Table 2:** The dinucleotide percentages (ratios)

Dinucleotdes	Ratios
AA	0.1
AC	0.09
AG	0.05
AT	0.07
CA	0.09
CC	0.11
CG	0.03
CT	0.09
GA	0.04
GC	0.04
GG	0.03
GT	0.03
TA	0.08
TC	0.07
TG	0.03
TT	0.06

**Table 3:** Best performance and regression values for 1-gram with varying number of neurons in the hidden layer

No. of neurons	Best perf. Values	Training	Validation	Testing
20	0.003209	0.99202	0.99054	0.97872
25	0.003173	0.99788	0.99055	0.9808
30	0.003137	0.99211	0.9907	0.97821
40	0.003195	0.99205	0.99049	0.97869
Averages	0.003178	0.99194	0.99057	0.97911

**Table 4:** Best performance and Regression values for 2-gram with varying number of neurons in the hidden layer

No. of neurons	Best perf. Values	Training	Validation	Testing
20	0.027319	0.94319	0.91605	0.88381
25	0.025913	0.9383	0.93036	0.90905
30	0.02278	0.9357	0.9302	0.90725
40	0.022379	0.93779	0.93157	0.89763
Averages	0.024598	0.93875	0.92705	0.89944

**Table 5:** Best performance and Regression values with 1-2-gram with varying number of neurons in the hidden layer

No. of neurons	Best perf. values	Training	Validation	Testing
20	0.002849	0.99378	0.99148	0.9801
25	0.002666	0.99395	0.99212	0.98136
30	0.00313	0.9942	0.99078	0.98123
40	0.002525	0.99388	0.98245	0.98128
Averages	0.002793	0.99395	0.99171	0.98099
